# The impacts of single nucleotide polymorphisms in genes of cell cycle and NF-κB pathways on the efficacy and acute toxicities of radiotherapy in patients with nasopharyngeal carcinoma

**DOI:** 10.18632/oncotarget.15835

**Published:** 2017-03-02

**Authors:** Chengxian Guo, Yuling Huang, Jingjing Yu, Lijuan Liu, Xiaochang Gong, Min Huang, Chunling Jiang, Yulu Liao, Lihua Huang, Guoping Yang, Jingao Li

**Affiliations:** ^1^ Center of Clinical Pharmacology, The Third Xiangya Hospital, Central South University, Changsha, Hunan 410013, China; ^2^ Department of Radiation Oncology, Jiangxi Cancer Hospital, Nanchang, Jiangxi 330029, China; ^3^ Department of Pharmacy, Jiangxi Cancer Hospital, Nanchang, Jiangxi 330029, China; ^4^ Center for Medical Experiments, The Third Xiangya Hospital, Central South University, Changsha, Hunan 410013, China

**Keywords:** nasopharyngeal carcinoma (NPC), cell cycle, NF-κB pathway, single nucleotide polymorphism (SNP), clinical responses

## Abstract

Radiotherapy is one of the primary choices for the treatment of nasopharyngeal carcinoma (NPC) and may result in severe radiotoxicities on normal tissues. Single nucleotide polymorphisms (SNPs) in genes of cell cycle and NF-κB pathways have been linked with the prognoses of various cancers. The aim of this study was to explore whether SNPs of genes involved in cell cycle and NF-κB pathways are associated with responses to radiotherapy in NPC patients. We selected 3 SNPs in cell cycle pathway and 5 SNPs in NF-κB pathway and genotyped them in 154 NPC patients treated with radiotherapy. Multivariate logistic regression was used to determine the association of these 8 SNPs with the responses to radiotherapy. We observed that cyclin-dependent kinase inhibitor gene *CDKN2A* rs3088440 was significantly related with a poorer treatment efficacy on the primary tumor and cervical lymph node after radiotherapy, and also with a decreased risk of grade 3–4 acute radiation-induced myelosuppression. In some subgroups, cyclin D1 gene *CCND1* rs9344 and inhibitor of κB kinase gene *IKBKB* rs12676482 were related with the grade 3–4 acute radiation-induced myelosuppression, and *CCND1* rs9344 was also associated with grade 3–4 acute radiation-induced oral mucositis. The current results reveal that SNPs in genes of cell cycle pathwayand NF-κB pathway have the potential to predict the clinical responses to radiotherapy for NPC patients.

## INTRODUCTION

Nasopharyngeal carcinoma (NPC) is one of the common head and neck cancers originating from the epithelial lining of the nasopharynx [1]. With a notably ethnically and geographically distributed imbalance, the incidence of NPC is much higher in southern China than Western countries [2]. Radiotherapy is one of the primary choices for the treatment of NPC due to the deep-rooted histological location and relative radiosensitivity of the carcinoma [3]. Despite the development of advanced radiotherapy techniques such as intensity-modulated radiotherapy (IMRT), a certain number of patients may experience radioresistance, which causes the distant metastasis or recurrence of the tumor. Since radiation may effect on normal tissues, patients treated with radiotherapy often suffer some complications like dermatitis, oral mucositis, dysphagia and hoarseness, which severely reduce the patients’ life quality and even reduce the treatment efficacy due to frequent dose adjustment and delays of radiotherapy [4, 5]. Moreover, the clinical outcome and the incidence of complications are heterogeneous among patients, which can be attributed to not only demographic and environmental factors, but also genetic factors [6].

With the rapid development of radiogenomics, a number of studies have illuminated the significant association between genetic variants with the efficacy and toxicity of radiotherapy in patients with NPC [7–12]. For example, Cheuk's preliminary results indicated that *XRCC3* rs861539 polymorphism may be associated with increased risk of radiation-induced fibrosis in NPC patients [13]. Moreover, Li et al. have found that *XRCC1* polymorphism affecting 399Gln is related with radiation-induced dermatitis and mucositis in NPC patients, and carriers of *XRCC1* 399Gln/Arg coding genotype had a higher risk of severe acute dermatitis and oral mucositis [14].

Cell cycle is the most essential physiological process, and dysregulation of normal cell cycle control has been implicated in the pathogenesis of most human cancers [15]. Main proteins involved in cell cycle regulation are cyclins, cyclin-dependent kinases (CDKs) and cyclin-dependent kinase inhibitors (CDKNs) [16]. Cyclin D1 encoded by the *CCND1* gene is a key positive regulatory protein of the G1/S phase in the cell cycle. Variations of *CCND1* have been detected in many cancers and *CCND1* rs9344 polymorphism has been frequently reported to be related with several cancers including NPC [17–20]. One study found that the G allele of *CCND1* rs9344 polymorphism was associated with a decreased risk of developing NPC [21]. The cyclin-dependent kinase inhibitor p21 encoded by *CDKN1A* gene inhibits the phosphorylation of Retinoblastoma protein (Rb) by binding to cyclin-cdk complexes: cyclinE-cdk2, cyclinA-cdk2, and cyclinD-cdk4 [22]. *CDKN1A* rs1059234 polymorphism was found to be associated with increased risk of head and neck squamous cell carcinoma (HNSCC) [23]. Another tumor suppressor gene, *CDKN2A*, encoding two proteins p14ARF and p16INK4a, has also been reported to be associated with the development of several cancers, such as HNSCC [24, 25], cervical neoplasia [26] and glioma [27].

In addition, nuclear factor-kappa B (NF-κB) has also been involved with cancer biology through regulation of proliferation factors such as CCND1 [28]. Some SNPs of genes involved in NF-κB signaling pathway have been frequently found associated with several cancers [29, 30]. For example, Hein et al. reported that individuals with two minor alleles of SNP rs1054016 of *RANK* gene (coding the receptor activator of NF-κB) had a more favorable prognosis than those with at least one common allele in patients with breast cancer [31]. Recently, one study found that multiple loss-of-function mutations were identified in several NF-κB signaling negative regulators tumor necrosis factor a-induced protein 3 (*TNFAIP3*), NF-κB inhibitor alpha (*NFKBIA*), and cylindromatosis (*CYLD*) in NPC. And the mutations in *NFKBIA* had a noticeable impact on NPC cell growth, which revealed the association between NF-κB signaling pathway and NPC [32]. Canonical NF-κB signaling is performed by a series of positive regulators such as tumor necrosis factor receptor-associated factor-6 (TRAF6) and negative regulators such as TNFAIP3 interacting protein 1 (TNIP1). Inhibitor of κB kinase (IKK) β encoded by *IKBKB* gene is a kinase subunit of the IKK complex and plays a vital role in the activation of canonical NF-κB signaling pathway [33]. *NF- κBIL1* gene encodes the NF-κB inhibitor-like protein 1 that represents a novel member of the inhibitor of κB proteins (IκBs) family, which prevent the nuclear translocation of NF-κB [34]. TNIP1 inhibits NF-κB signaling pathway by cooperating with TNFAIP3 [35]. Some SNPs of aforementioned genes involved in NF-κB signaling pathway have been identified and linked with several diseases [36–39].

However, there has been no study that explores whether genetic polymorphisms of aforementioned cell cycle regulation and NF-κB signaling related genes are associated with the sensibility to radiotherapy in NPC patients. We hypothesized that some potentially functional SNPs of genes in cell cycle pathway and NF-κB pathway might have prognostic values for NPC patients treated with radiotherapy. According to previously published literatures, we chose 3 SNPs (*CCND1* rs9344, *CDKN1A* rs1059234 and *CDKN2A* rs3088440) in cell cycle pathway and 5 SNPs (*IKBKB* rs12676482, *TRAF6* rs4755453 and rs5030437, *TNIP1* rs10036748 and *IκBL* rs2071592) in NF-κB pathway. The aim of this present study is to evaluate the association of the 8 SNPs with the efficacy and acute toxic reactions after radiotherapy in NPC patients and to find some novel genetic markers for the prognosis of NPC patients treated with radiotherapy.

## RESULTS

### Clinical characteristics, radiotherapy responses and genotyping of the patients

This study population consisted of 106 males and 48 males, with a mean age of 51 (ranging from 14 to 81). Thirty-two (20.8%) patients were treated with radiotherapy alone, and 122 patients (79.2%) were also treated with chemoradiotherapy. The general demographics and clinic pathologic characteristics as well as the clinical outcomes after radiotherapy of the 154 patients with NPC are summarized in Table 1. Because of some data missing, there were only data of 125 patients for curative efficacy at the cervical lymph node after radiotherapy. Overall, there were 29 (18.8%) and 17 (13.6%) patients who did not get CR after radiotherapy at their primary tumors and cervical lymph nodes, respectively. In the case of toxic reactions, 6 (3.9%), 71 (46.1%) and 32 (20.8%) patients experienced grade 3–4 acute radiation-induced dermatitis, oral mucositis, and myelosuppression, respectively. The associations of all clinical factors with the responses to radiotherapy are shown in Supplementary Tables 1 and 2. Patients getting CR at the cervical lymph node were significantly older than those not getting CR (*P* = 0.046). And compared with radiotherapy alone, patients treated with chemoradiotherapy had a higher risk of grade 3–4 myelosuppresion (*P* = 0.006). The results of genotyping and the allele type of each SNP in the 154 patients are shown in Table 2. All minor allele frequencies (MAFs) of 8 SNPs were > 0.10 and all genotype distributions were in accordance with Hardy-Weinberg equilibrium.

**Table 1:** The characteristics and clinical outcomes of the patients

Characteristics	Patients (%) (n=154)
Gender	
male	106 (68.8)
female	48 (31.2)
Age	
range	14 – 81
mean ± SD	51 ± 12
BMI	
range	16.90 – 32.00
mean ± SD	22.79 ± 3.27
Smoking	
yes	70 (45.5)
no	84 (54.5)
Drinking	
yes	38 (24.7%)
no	116 (75.3%)
Chemotherapy	
no	32 (20.8%)
yes	122 (79.2%)
Non-CR after radiotherapy	
primary tumor	29 (18.8%)
lymph node	17 (13.6%)
Grade 3–4 radiation-induced toxic reactions	
dermatitis	6 (3.9%)
oral mucositis	71 (46.1%)
myelosuppression	32 (20.8%)

Abbreviations: BMI, body mass index; CR, complete remission.

**Table 2:** Genotyping and MAF of 8 SNPs in genes of cell cycle and NF-κB pathways

SNP	Location	Alleles(mutant/wild)	N(AA/Aa/aa)	*P* (HWE)	MAF
*CCND1* rs9344	Extron	G/A	23/86/45	0.08	0.43
*CDKN2A* rs3088440	Downstream 500B	A/G	35/80/39	0.62	0.49
*CDKN1A* rs1059234	3′UTR	C/T	2/32/121	0.94	0.12
*IKBKB* rs12676482	Intron	A/G	1/34/119	0.39	0.12
*TRAF6* rs4755453	Upstream 2KB	C/G	2/35/117	0.73	0.13
*TNIP1* rs10036748	Intron	C/T	8/61/85	0.49	0.25
*NF-KBIL1* rs2071592	Promoter	T/A	37/68/49	0.17	0.46
*TRAF6* rs5030437	Intron	A/G	1/33/120	0.43	0.11

AA/Aa/aa:wild type/heterozygote/mutant homozygote;

Abbreviations: HWE, Hardy-Weinberg equilibrium; MAF, minor allele frequency; UTR, untranslated regions.

### Associations between 8 SNPs and the efficacy of radiotherapy

The associations between selected 8 SNPs and the efficacy at the primary tumor and cervical lymph node after radiotherapy are shown in Tables 3 and 4, respectively. *CDKN2A* rs3088440 polymorphism was significantly associated with poorer efficacy at the primary tumor and cervical lymph node after radiotherapy in multiplicative, additive and dominant models after adjusted by age, gender, BMI, smoking and drinking status, TNM stage and with/without chemotherapy. In detail, compared with the G allele, patients with the minor A allele of this SNP had a 2-fold and 3-fold higher risk to get non-CR at the primary tumor (OR: 2.20, *P*: 0.043) and at the cervical lymph node (OR: 3.04, *P*: 0.019) after radiotherapy, respectively. Another SNP, *NF-KBIL1* rs2071592, was related with a favorable outcome at the cervical lymph node in multiplicative (OR: 0.46, *P*: 0.011) and additive models (OR: 0.53, *P*: 0.046).

**Table 3:** The associations between 8 SNPs and the efficacy of radiotherapy for the primary tumor

SNP	Alleles^a^	MAF		N^b^		Multiplicative		Additive		Dominant		Recessive	
		n-CR	CR	n-CR	CR	OR(95% CI)	*P*	OR(95% CI)	*P*	OR(95% CI)	*P*	OR(95% CI)	*P*
rs9344	G/A	0.38	0.44	1/20/8	22/66/37	0.78(0.43-1.40)	0.400	0.82(0.43-1.57)	0.547	0.55(0.23-1.32)	0.179	0.14(0.02-1.16)	0.069
rs3088440	A/G	0.19	0.10	0/11/18	2/20/103	2.20(1.01-4.81)	**0.043**	2.37(1.03-5.45)	**0.043**	0.21(0.04-0.96)	**0.044**	NA	NA
rs1059234	C/T	0.52	0.48	8/14/7	27/66/32	1.16(0.66-2.06)	0.609	1.09(0.60-1.99)	0.781	0.64(0.26-1.59)	0.339	1.22(0.47-3.19)	0.682
rs12676482	A/G	0.10	0.12	0/6/23	1/28/96	0.85(0.33-2.14)	0.724	0.72(0.26-1.96)	0.519	1.34(0.52-3.44)	0.542	NA	NA
rs4755453	C/G	0.12	0.13	0/7/22	2/28/95	0.94(0.39-2.24)	0.880	1.14(0.45-2.89)	0.788	0.64(0.23-1.78)	0.393	NA	NA
rs10036748	C/T	0.29	0.24	3/11/15	5/50/70	1.31(0.70-2.48)	0.400	1.25(0.63-2.49)	0.521	0.45(0.19-1.06)	0.068	2.75(0.52-14.46)	0.234
rs2071592	T/A	0.31	0.50	3/12/14	34/56/35	0.46(0.25-0.84)	**0.011**	0.53(0.29-0.99)	**0.046**	0.82(0.33-2.00)	0.660	0.33(0.09-1.23)	0.099
rs5030437	A/G	0.14	0.11	0/8/21	1/25/99	1.32(0.57-3.08)	0.518	1.52(0.60-3.86)	0.380	0.45(0.14-1.42)	0.171	NA	NA

^a^ In the order of mutant/wild; ^b^In the order of mutant homozygote/heterozygote/wild homozygote;

Abbreviations: CR, complete remission; MAF, minor allele frequency; OR, odds ratio; CI, confidence interval; NA, not applicative. *P* value < 0.05 is shown in bold.

**Table 4:** The associations between 8 SNPs and the efficacy of radiotherapy for the cervical lymph node

SNP	Alleles^a^	MAF		N ^b^		multiplicative		additive		dominant		recessive	
		n-CR	CR	n-CR	CR	OR(95%CI)	P	OR(95%CI)	P	OR(95%CI)	P	OR(95%CI)	P
rs9344	G/A	0.35	0.42	2/8/7	14/63/31	0.75(0.35-1.59)	0.452	0.79(0.34-1.82)	0.583	0.70(0.23-2.10)	0.523	0.87(0.17-4.52)	0.866
rs3088440	A/G	0.21	0.08	0/7/10	1/15/92	3.04(1.15-7.99)	**0.019**	3.58(1.22-10.52)	**0.021**	4.69(1.44-15.3)	**0.011**	NA	NA
rs1059234	C/T	0.53	0.50	5/8/4	24/61/23	1.10(0.54-2.28)	0.788	1.03(0.47-2.23)	0.947	0.90(0.25-3.21)	0.877	1.17(0.35-3.98)	0.797
rs12676482	A/G	0.15	0.13	0/5/12	1/27/80	1.11(0.40-3.10)	0.840	1.01(0.32-3.17)	0.992	1.05(0.32-3.44)	0.939	NA	NA
rs4755453	C/G	0.12	0.12	1/2/14	1/24/83	0.97(0.32-2.99)	0.964	1.10(0.34-3.60)	0.875	0.76(0.19-3.05)	0.703	15.2(0.69-336.20)	0.085
rs10036748	C/T	0.29	0.26	1/8/8	5/46/57	1.19(0.54-2.64)	0.668	1.13(0.46-2.77)	0.796	1.10(0.38-3.17)	0.860	1.44(0.14-15.13)	0.764
rs2071592	T/A	0.29	0.46	1/8/8	26/47/35	0.49(0.22-1.08)	0.073	0.49(0.22-1.12)	0.093	0.54(0.18-1.64)	0.273	0.16(0.02-1.45)	0.104
rs5030437	A/G	0.06	0.12	0/2/15	1/24/83	0.46(0.10-2.02)	0.290	0.45(0.09-2.16)	0.320	0.45(0.09-2.21)	0.327	NA	NA

^a^ In the order of mutant/wild; ^b^In the order of mutant homozygote /heterozygote/ wild homozygote;

Abbreviations: CR, complete remission; MAF, minor allele frequency; OR, odds ratio; CI, confidence interval; NA, not applicative.

*P* value < 0.05 is shown in bold.

### Stratification analyses of the associations between 8 SNPs and the efficacy of radiotherapy

We performed stratification analyses to evaluate the impact of selected 8 SNPs on the efficacy of radiotherapy in NPC patients sorted by each clinical factor. As shown in Table 5, *CDKN2A* rs3088440 was significantly associated with the efficacy at the primary tumor and cervical lymph node among patients ≥ 51 years old, patients with BMI ≥ 22.8, non-smoking patients and patients treated with chemoradiotherapy. *NF-KBIL1* rs2071592 was related with the efficacy at the primary tumor among patients ≥ 51 years old, patients with BMI < 22.8, smoking patients and patients treated with chemoradiotherapy, and this SNP was related with the efficacy at the cervical lymph node in smoking patients in multiplicative and additive models. In addition, *TRAF6* rs5030437 (among patients ≥ 51 years old) and *IKBKB* rs12676482 (among patients with BMI < 22.8) were associated with the efficacy at the primary tumor in multiplicative, additive, and dominant models.

**Table 5:** Stratification analysis of association between 8 SNPs and the efficacy of radiotherapy in NPC patients

SNPs (Alleles^a^)	Subgroups (n)	Multiplicative		Additive		Dominant		Recessive	
		OR(95%CI)	*P*	OR(95%CI)	*P*	OR(95%CI)	*P*	OR(95%CI)	*P*
**At the primary tumor**									
rs3088440(A/G)	≥ 51 y (80)	4.40(1.58-12.28)	**0.003**	5.04(1.59-16.04)	**0.006**	7.47(2.13-26.17)	**0.002**	NA	
	High BMI (68)	4.57(1.41-14.82)	**0.007**	4.50(1.31-15.5)	**0.017**	7.31(1.89-28.23)	**0.004**	NA	
	Non-smoker (84)	2.41(0.87-6.67)	0.082	2.48(0.86-7.13)	0.092	3.18(1.01-10.03)	**0.048**	NA	
	CRT(122)	2.11(0.85-5.22)	0.102	2.02(0.83-4.93)	0.122	2.77(1.01-7.61)	**0.049**	NA	
rs2071592(T/A)	≥ 51 y (80)	0.38(0.15-0.92)	**0.027**	0.4(0.17-0.97)	**0.042**	0.50(0.15-1.64)	0.253	NA	
	Low BMI(86)	2.73(1.12-6.67)	**0.024**	2.75(1.09-6.91)	**0.032**	2.22(0.45-10.89)	0.325	4.92(1.43-16.93)	**0.012**
	Smoker (70)	3.16(1.16-8.64)	**0.020**	3.20(1.13-9.09)	**0.029**	NA		2.95(0.77-11.23)	0.113
	CRT(122)	0.45(0.23-0.87)	**0.017**	0.50(0.26-0.94)	**0.031**	0.40(0.16-1.00)	**0.049**	0.34(0.09-1.23)	0.100
rs5030437 (A/G)	≥ 51 y (80)	2.81(1.01-7.79)	**0.040**	3.07(1.02-9.29)	**0.047**	4.08(1.22-13.68)	**0.023**	NA	
rs12676482(A/G)	Low BMI(86)	2.83(0.97-8.21)	**0.048**	3.61(1.05-12.44)	**0.042**	3.61(1.05-12.44)	**0.042**	NA	
**At the cervical lymph node**									
rs3088440(A/G)	≥51 y (62)	3.75(1.11-12.68)	**0.025**	4.67(1.22-17.84)	**0.024**	4.67(1.22-17.84)	**0.024**	NA	
	High BMI (56)	4.20(0.9-19.69)	0.052	3.36(0.77-14.75)	0.108	6.6(1.14-38.35)	0.036	NA	
	Non-smoker (67)	4.42(1.28-15.28)	**0.012**	6.13(1.44-26.05)	**0.014**	6.13(1.44-26.05)	**0.014**	NA	
	CRT(105)	3.21(1.12-9.25)	**0.024**	3.25(1.08-9.76)	**0.036**	4.33(1.31-14.37)	**0.016**	NA	
rs2071592(T/A)	Smoker (58)	3.97(1.04-15.06)	**0.032**	4.00(1.01-15.85)	**0.049**	NA		4.33(0.85-22.13)	0.078

^a^ In the order of mutant/wild;

Abbreviations: CRT, chemoradiotherapy; BMI, body mass index; OR, odds ratio; CI, confidence interval; NA, not applicable.

High BMI: BMI ≥ 22.8; Low BMI: BMI < 22.8; *P* value < 0.05 is shown in bold.

### Associations between the 8 SNPs and grade 3–4 acute radiation-induced toxic reactions

The associations between the selected 8 SNPs and grade 3–4 acute radiation-induced myelosuppression are shown in Table 6. *CDKN2A* rs3088440 polymorphism was significantly correlated with grade 3–4 acute radiation-induced myelosuppression in multiplicative (OR: 0.21, *P*: 0.020), additive (OR: 0.22, *P*: 0.045) and dominant (OR: 0.21, *P*: 0.044) models after adjustment. Patients carrying the minor A allele of rs3088440 were more resistant to acute radiation-induced myelosuppression. None of the 8 SNPs was found significantly associated with acute radiation-induced dermatitis and oral mucositis after adjustment (Supplementary Tables 3 and 4).

**Table 6:** The associations between 8 SNPs and the grade 3–4 acute radiation-induced myelosuppression

SNP	Alleles^a^	MAF		N^b^		multiplicative		additive		dominant		recessive	
		G3+	G0-2	G3+	G0-2	OR(95% CI)	*P*	OR(95% CI)	*P*	OR(95% CI)	*P*	OR(95% CI)	*P*
rs9344	G/A	0.38	0.44	5/14/13	18/72/32	0.76(0.43-1.33)	0.331	0.74(0.39-1.39)	0.347	0.55(0.23-1.32)	0.179	0.99(0.32-3.05)	0.982
rs3088440	A/G	0.03	0.14	0/2/30	2/29/91	0.21(0.05-0.88)	0.020	0.22(0.05-0.97)	0.045	0.21(0.04-0.96)	0.044	NA	NA
rs1059234	C/T	0.47	0.49	8/14/10	27/66/29	0.91(0.53-1.58)	0.743	0.81(0.45-1.45)	0.473	0.64(0.26-1.59)	0.339	0.9(0.34-2.38)	0.840
rs12676482	A/G	0.16	0.11	1/8/23	0/26/96	1.55(0.71-3.41)	0.271	1.55(0.64-3.77)	0.330	1.34(0.52-3.44)	0.542	NA	NA
rs4755453	C/G	0.09	0.14	0/6/26	2/29/91	0.66(0.26-1.66)	0.374	0.60(0.23-1.56)	0.300	0.64(0.23-1.78)	0.393	NA	NA
rs10036748	C/T	0.17	0.27	1/9/22	7/52/63	0.56(0.28-1.14)	0.105	0.52(0.24-1.16)	0.112	0.45(0.19-1.06)	0.068	1.28(0.12-13.79)	0.840
rs2071592	T/A	0.48	0.45	10/11/11	27/57/38	1.13(0.65-1.95)	0.674	0.99(0.57-1.73)	0.982	0.82(0.33-2.00)	0.660	1.22(0.48-3.1)	0.672
rs5030437	A/G	0.06	0.13	0/4/28	1/29/92	0.46(0.16-1.35)	0.148	0.44(0.14-1.36)	0.156	0.45(0.14-1.42)	0.171	NA	NA

^a^ In the order of mutant/wild; ^b^In the order of mutant homozygote /heterozygote/ wild homozygote;

Abbreviations: CR, complete remission; MAF, minor allele frequency; G, grade; OR, odds ratio; CI, confidence interval; NA, not applicative. *P* value < 0.05 is shown in bold.

### Stratification analyses of the associations between 8 SNPs and grade 3–4 acute radiation-induced toxic reactions

As shown in Table 7, *CCND1* rs9344 was related with grade 3–4 acute radiation-induced oral mucositis in recessive model among patients < 51 years old, and it was also related with grade 3–4 acute radiation-induced myelosuppression in additive and recessive models among patients with BMI < 22.8. *CDKN2A* rs3088440 polymorphism was correlated with grade 3–4 acute radiation-induced myelosuppression among III-IV stage patients and patients treated with chemoradiotherapy. *IKBKB* rs12676482 was associated with grade 3–4 acute radiation-induced myelosuppression in multiplicative and additive models among non-smoking patients. Individuals carrying the minor A allele of rs9344 and rs3088440 were less sensitive to the radiation-induced toxicity, while patients with the minor A allele of rs12676482 had a higher risk of grade 3–4 acute radiation-induced myelosuppression, compared with the G allele.

**Table 7:** Stratification analysis of association between SNPs and the acute radiation-induced toxic reactions in NPC patients

SNPs (Alleles^a^)	Subgroups (n)	Multiplicative		Additive		Dominant		Recessive	
		OR(95%CI)	*P*	OR(95%CI)	*P*	OR(95%CI)	*P*	OR(95%CI)	*P*
Grade 3–4 oral mucositis									
rs9344 (G/A)	< 51 y(74)	0.65(0.33-1.27)	0.208	0.65(0.33-1.28)	0.211	1.15(0.45-2.96)	0.766	0.08(0.01-0.66)	**0.019**
Grade 3–4 myelosuppression									
rs3088440 (A/G)	III-IV stage(131)	0.24(0.06-1.06)	**0.043**	0.25(0.06-1.09)	**0.065**	0.24(0.05-1.09)	0.064	NA	
	CRT(122)	0.23(0.05-1.01)	**0.035**	0.24(0.06-1.05)	**0.058**	0.23(0.05-1.04)	0.057	NA	
rs9344 (G/A)	Low BMI(86)	0.49(0.23-1.05)	0.064	0.42(0.18-1.00)	**0.049**	0.29(0.1-0.86)	**0.026**	NA	
rs12676482(A/G)	Non-smoker(84)	2.57(1.01-6.52)	**0.042**	2.83(1.03-7.77)	**0.043**	2.65(0.89-7.85)	0.079	NA	

^a^ In the order of mutant/wild;

Abbreviations: CRT, chemoradiotherapy; BMI, body mass index; OR, odds ratio; CI, confidence interval; NA, not applicable.

High BMI: BMI ≥ 22.8; Low BMI: BMI < 22.8; *P* value < 0.05 is shown in bold.

## DISCUSSION

Deregulation of the cell cycle is one of the most frequent alterations in the process of tumor development, and genetic polymorphisms of genes involved in cell cycle control have been implicated in development and prognosis of many cancers. The NF-κB signaling pathway has also been linked with several cancers by regulating transcription of its downstream target genes, including genes involved in cell cycle pathway [28, 40]. Many studies have found that NF-κB signaling was exceedingly activated and some target genes of NF-κB were upregulated in several cancers [41–43]. As far as we know, this is the first time to investigate the impact of SNPs in the cell cycle pathway and NF-κB signaling pathway genes on the efficacy and toxicity of radiotherapy in NPC patients treated with radiotherapy (or chemoradiotherapy). Our results showed that 4 SNPs (*CDKN2A* rs3088440, *NF-KBIL1* rs2071592, *TRAF6* rs5030437 and *IKBKB* rs12676482) were related with the efficacy of radiotherapy and 3 SNPs (*CDKN2A* rs3088440, *CCND1* rs9344 and *IKBKB* rs12676482) were related with the acute radiation-induced toxic reactions in NPC patients.

The results of logistic regressions in whole patients and some subgroups indicated that *CDKN2A* rs3088440 polymorphism was markedly associated with a poorer efficacy of radiotherapy at the primary tumor and cervical lymph node, as well with a decreased risk of grade 3–4 acute radiation-induced myelosuppression. This revealed that the mutant A allele of rs3088440 was related with a more robust resistance to radiotherapy than the G allele. Rs3088440 was identified in the 3′-UTR of *CDKN2A* and has been reported to be relevant to the susceptibility and prognosis of several cancers in spite of inconsistent results in previous studies [44–46]. The association between rs3088440 polymorphism and the increased risk of HPV16-positive oropharynx cancer has also been reported, while no significant prognostic effect of rs3088440 polymorphism was found on the survival after definitive chemoradiotherapy in HPV16-positive oropharynx cancer patients [45]. Two previous studies have reported the significant relation between the A allele of *CDKN2A* rs3088440 polymorphism and the higher risk of melanoma [46, 47], but other studies could not replicate such result [48, 49]. In another separate study, *CDKN2A* rs3088440 polymorphism was associated with reduced risk of progression from Barrett's esophagus to esophageal adenocarcinoma [50]. The result of their *in vitro* functional studies suggested that the transcript of *CDKN2A* may be suppressed by miR-663b in a rs3088440 G allele-dependent manner. Under that circumstance, the rs3088440 A mutant would potentially impair the binding of miR-663b to the *CDKN2A* 3′ UTR and lead to enhanced expression of the p14/p16 proteins. The results of the present study suggested a potential mechanism that the enhancement of *CDKN2A* expression due to the G>A substitution results in the proliferation of NPC cells, which ulteriorly weakens the treatment effect of radiotherapy and alleviates the radiation-induced toxicity.

We also found that the AA genotype of *CCND1* rs9344 was associated with a decreased risk of grade 3–4 oral mucositis among patients < 51 years old. And among patients with BMI < 22.8, this SNP was related with grade 3–4 myelosuppression in multiplicative (OR: 0.42, *P*: 0.049) and additive (OR: 0.29, *P*: 0.026) models. The G>A mutant of *CCND1* rs9344 changes the splicing of exon 4 and then alters the *CCND1* expression, which may promote its oncogenic potential [51]. Previous studies mostly investigated the impact of rs9344 on the susceptibility and survival of many cancers, such as breast cancer, NPC, HCC and oral cancer [21, 52, 53]. However, there has been no study exploring the influence of this SNP on the toxicity of radiotherapy. According to this present study, the A allele of *CCND1* rs9344 was associated with a more robust resistance to radiotherapy and the result needs more large-size studies to duplicate.

In addition, *NF-KBIL1* rs2071592 was related with a more favorable outcome at the primary tumor in the multivariate logistic regression as well as among patients ≥ 51 years old and treated with chemoradiotherapy. But on the contrary, rs2071592 was linked with a poorer outcome at the primary tumor among patients with BMI < 22.8 and smoking patients. NF-KBIL1 is a novel inhibitor of NF-κB signaling and the polymorphism rs2071592 in its gene promoter region has been reported to be significantly associated with rheumatoid arthritis (RA) [54] and systemic lupus erythematosus (SLE) [34]. The A allele of this SNP is presumed to disrupt the binding of transcriptional factor and affect the expression of *NF-KBIL1*. Since our contradictory results, the authentic relationship between *NF-KBIL1* rs2071592 with the efficacy of radiotherapy in NPC remains uncertain and the exact mechanism needs more studies to explain.

In the subgroup analysis, *TRAF6* rs5030437 and *IKBKB* rs12676482 were associated with less favorable outcome at the primary tumor in patients ≧ 51 years old and patients with BMI < 22.8, respectively. TRAF6 and IKBKB are positive regulators of NF-κB signaling, of which rs5030437 and rs12676482 are both identified in the intron region of corresponding gene. These two SNPs may upregulate the gene expression by affecting mRNA splicing, localization or stability, and reinforce the NF-κB signaling, which facilitates the proliferation of NPC cells and weakens the effect of radiotherapy [55]. Another possible mechanism is that the rs5030437 or rs12676482 is in linkage disequilibrium (LD) with a certain functional variant that really mediates this process [56]. Whatever, there has no report about the correlation between rs5030437 and rs12676482 polymorphisms with the prognosis of NPC, and more studies are requisite to verify the specific mechanism.

The obvious advantage of our study lies in that this is the first time to assess the association of SNPs in cell cycle pathway and NF-κB signaling pathway genes with clinical responses to radiotherapy in NPC patients. However, our study still had some limitations. First, the small sample size reduced the statistical power, especially the limited numbers of patients in some subgroups, which resulted in the inconsistent results for certain findings in different subgroups. Second, the mechanisms that how these SNPS influenced the treatment efficacy and toxicity of radiotherapy in NPC patients were not fully understood, as those were beyond the scope of the present study. Third, we did not detect the levels of corresponding mRNA and protein expression of each studied gene, so the findings in this study still remain to be confirmed in more researches.

## MATERIALS AND METHODS

### Study population

This study collected 154 NPC patients treated with radiotherapy (with or without joint chemotherapy) from the Jiangxi Cancer Hospital from April 2014 to September 2015. The patients were eligible for this study if they met the following criteria: pathologically confirmed NPC; no distant metastasis; performance status score ≤ 1; no severe dysfunction of lung, heart, liver and kidney; no treatment with any anticancer therapies before radiotherapy; and signing the informed consent before this study. Exclusion criteria included: pregnancy or lactation; active infection before radiotherapy; and previous or concomitant malignancies. We recorded all demographics and clinic pathologic data of 154 patients including age, gender, body mass index (BMI), smoking and drinking status, TNM (7^th^ UICC) stage and with/without chemotherapy. The study was approved by the Ethics Committee of Cancer Hospital of Jiangxi Province (China) and we applied for clinical admission for this study through the Chinese Clinical Trial Registry (www.chictr.org.cn, registration number: ChiCTR-OPC-14005257).

### Efficacy regimen

All selected patients were treated with IMRT technique. Most patients (122) were treated with IMRT as well as chemotherapy while the other 32 were treated with IMRT alone. For nasopharyngeal primary focus and cervical positive lymph nodes, the total dose of radiotherapy was 66–70 Gy in 30–33 fractions, and for cervical drainage region, patients received 54–60 Gy in all in 30–33 fractions. One fraction was administrated daily and five fractions per week. The inductive chemotherapy and concurrent chemotherapy referred to platinum-based chemotherapy regimens.

### Evaluation on the radiotherapy treatment efficacy and toxic reactions

The curative efficacy at the primary tumor and cervical lymph node was evaluated by magnetic resonance imaging (MRI) directly after finishing radiotherapy. According to the Response Evaluation Criteria In Solid Tumors (RECIST) of World Health Organization (WHO), the efficacy was defined as complete remission (CR), partial remission (PR), stable disease (SD) or progression disease (PD) based on the tumor volume. All patients of this study were subdivided into two groups: CR, and non-CR consisting of PR, SD and PD for the efficacy of primary tumor and cervical lymph node.

We observed and recorded acute radiation-induced toxic reactions, including myelosuppression, dermatitis and oral mucositis, once a day from the first day to the end of radiotherapy. All toxic reactions were evaluated and classified as grade 0-4 based on the acute radiation toxicity grading criterion of the Radiation Therapy Oncology Group or European Organization for Research and Treatment of Cancer (RTOG/EORTC). According to the grading results, we divided the patients into two groups: non-sensitive or mildly radiosensitive (grade 0–2), and highly radiosensitive (grade 3–4).

### DNA extraction and genotyping

Venous blood sample (3 mL) was taken from each patient before radiotherapy and stored in EDTA anticoagulated tubes at -20°C. Genomic DNA was extracted using Wizard Genomic DNA Purification Kit (Promega, Madison, USA). Genotyping for 8 SNPs was performed by Sequenom MassARRAY system (Sequenom, San Diego, California, USA). Primers were designed by using Assay Designer 3.1 and the polymerase chain reaction (PCR) was performed by following scheme: an initial denaturation for 15 min at 94°C, then 45 cycles of 20 s at 94°C, 30 s at 56°C, and 1 min at 72°C, and a final elongation for 3 min at 72°C. The products of PCR were purified using shrimp alkaline phosphatase (SAP) according to following process: initial incubation for 40 min at 37°C, followed by 85°C for 5 min. The purified products were extended by reactions using extension primers and subsequently desalted by the addition of a cation exchange resin. The desalinated products were transferred in CHIP after being centrifuged at 4000 rpm for 4 min. Data from CHIP were analyzed by Typer Analyzer.

### Statistical analysis

The association of each clinical factor with the efficacy or toxic reactions after radiotherapy was computed using chi-square test or t-test. Multivariate logistic regression was carried out to determine the associations between each SNP and non-CR of primary tumor and cervical lymph node as well as grade 3–4 toxic reactions, with CR and grade 0–2 as the reference, respectively. The odds ratio (OR) and the corresponding 95% confidence interval (CI) were computed after adjustment for age, gender, BMI, smoking and drinking habit, carcinoma stage, and with/without chemotherapy. All logistic regressions were conducted by four models: multiplicative, additive, dominant and recessive models, which were performed by PLINK 1.07 (https://www.cog-genomics.org/plink2). If X is the major allele and x is the minor allele, the analysis in multiplicative model breaks down the genotypes to compare the total numbers of X and x alleles of each SNP in two groups, regardless of the genotypes. The additive model is for the additive effect of each SNP, with a direction of the regression coefficient that represents the effect of each extra x allele. Dominant and recessive models are both tests for the minor allele with two of the three genotypes pooled. The dominant model means (xx+Xx) *versus* XX and the recessive model means xx *versus* (XX+Xx) [57]. In addition, stratification analyses were performed to determine the associations of SNPs and radiotherapy efficacy and toxic reactions in many subgroups sorted by age, gender, BMI, smoking and drinking habit, carcinoma stage, and with/without chemotherapy. The difference was considered statistically significant if *P* value < 0.05.

## SUPPLEMENTARY TABLES





## Abbreviations

NPC: nasopharyngeal carcinoma
IMRT: intensity modulated radiotherapy
CR: complete remission
PR: partial remission
SD: stable disease
PD: progression disease
SNP: single nucleotide polymorphism
CDK: cyclin-dependent kinase
CDKN: CDK inhibitor
CCND1: cyclin D1
NF-κB: nuclear factor-kappa B
HNSCC: head and neck squamous cell carcinoma
RANK: receptor activator of NF-κB
TNFAIP3: tumor necrosis factor alpha-induced protein 3
NFKBIA: NF-κB inhibitor alpha
CYLD: cylindromatosis
TRAF6: tumor necrosis factor receptor-associated factor-6
TNIP1: TNFAIP3 interacting protein 1
IκB: inhibitor of κB
IKK: inhibitor of κB kinase
NF-KBIL1: NF-κB inhibitor-like protein 1
MRI: magnetic resonance imaging
RECIST: response evaluation criteria in solid tumors
RTOG: radiation therapy oncology group
EORTC: European organization for research and efficacy of cancer
