# Supplementary material for: The impacts of single nucleotide polymorphisms in genes of cell cycle and NF-κB pathways on the efficacy and acute toxicities of radiotherapy in patients with nasopharyngeal carcinoma
Source: Oncotarget. 2017 Mar 2;8(15):25334–44. doi: 10.18632/oncotarget.15835 (PMC5421934; doi:10.18632/oncotarget.15835)
Supplement: Supplementary file 1 [file oncotarget-08-25334-s001.pdf]

The impacts of single nucleotide polymorphisms in genes of cell cycle and NF-κB pathways on the efficacy and acute toxicities of radiotherapy in patients with nasopharyngeal carcinoma

SUPPLEMENTRY TABLES

Supplementary Table 1: Association between clinical factors and the efficacy of RT directly after treatment and 3 months after treatment in patients with NPC

Clinical Characteristics	primary tumor (*n*=154)			lymph node (*n*=125)		
	CR	Non-CR	*P*	CR	Non-CR	*P*
Age	51.0 ± 11.4	48.1 ± 13.9	0.241	50.9 ± 12.0	44.6 ± 10.9	**0.042**
Gender						
Male	88(70.4)	18(62.1)	0.383	77(71.3)	10(58.8)	0.299
Female	37(29.6)	11(37.9)		31(28.7)	7(41.2)	
Drinking						
Yes	34(27.2)	4(13.8)	0.131	28(25.9)	3(17.6)	0.463
No	91(72.8)	25(86.2)		80(74.1)	14(82.4)	
Smoking						
Yes	59(47.2)	11(37.9)	0.366	57(52.8)	10(58.8)	0.642
No	66(52.8)	18(62.1)		51(47.2)	7(41.2)	
BMI	22.6 ± 3.1	23.8 ± 3.8	0.104	22.8 ± 3.2	23.0 ± 3.0	0.866
Chemotherapy						
No	27(21.6)	5(17.2)	0.602	18(16.7)	2(11.8)	0.608
Yes	98(78.4)	24(82.8)		90(83.3)	15(88.2)	
Clinical stage						
I–II	18(14.4)	5(17.2)	0.699	11(10.2)	2(11.8)	0.843
III–IV	107(85.6)	24(82.8)		97(89.8)	15(88.2)	

Abbreviations: BMI, body mass index. *P* value < 0.05 is shown in bold.

Supplementary Table 2: The association between clinical factors and the acute radiation-induced toxic reactions

Clinical characteristics	Dermatitis (*n*=154)			Oral mucositis (*n*=154)			Myelosuppression (*n*=154)		
	Grade 0–2	Grade 3–4	*P*	Grade 0–2	Grade 3–4	*P*	Grade 0–2	Grade 3–4	*P*
Age	50.7 ± 12.0	44.5 ±7.2	0.211	51.4 ± 12.2	49.4 ± 11.6	0.310	51.4 ± 11.7	47.0 ± 12.3	0.066
Gender									
male	101(68.2)	5(83.3)	0.739	56(67.5)	50(70.4)	0.693	85(69.7)	21(65.6)	0.660
female	47(31.8)	1(16.7)		27(32.5)	21(29.6)		37(30.3)	11(34.4)	
Drinking									
Yes	37(25.0)	1(16.7)	1.000	19(22.9)	19(26.8)	0.579	30(24.6)	8(25.0)	0.962
No	111(75.0)	5(83.3)		64(77.1)	52(73.2)		92(75.4)	24(75.0)	
Smoking									
Yes	66(44.6)	4(66.7)	0.518	36(43.3)	34(47.9)	0.575	57(46.7)	13(40.6)	0.538
No	82(55.4)	2(33.3)		47(56.6)	37(52.1)		65(53.3)	19(59.4)	
Family history									
Yes	23	2	0.922	14	11	0.719	19	6	0.940
No	149	14		85	78		125	38	
BMI	22.7 ± 3.2	24.2 ± 4.3	0.289	23.2 ± 3.2	22.4 ± 3.3	0.137	23.0 ± 3.3	22.1 ± 2.9	0.202
Chemotherapy									
No	31(20.9)	1(16.7)	1.000	21	17	0.719	31(25.4)	1(3.1)	**0.006**
Yes	117(79.1)	5(83.3)		78	72		91(74.6)	31(96.9)	
Clinical stage									
I–II	21(14.2)	2(33.3)	0.480	12(14.5)	11(15.5)	0.857	20(16.4)	3(9.4)	0.321
III–IV	127(85.8)	4(66.7)		71(85.5)	60(84.5)		102(83.6)	29(90.6)	

Abbreviations: BMI, body mass index. *P* value < 0.05 is shown in bold.

Supplementary Table 3: The associations between 8 SNPs and the grade 3–4 acute radiation-induced dermatitis

See Supplementary File 1

Supplementary Table 4: The associations between 8 SNPs and the grade 3–4 acute radiation-induced oral mucositis

See Supplementary File 1
