# Supplementary material for: The impacts of single nucleotide polymorphisms in genes of cell cycle and NF-κB pathways on the efficacy and acute toxicities of radiotherapy in patients with nasopharyngeal carcinoma
Source: Oncotarget. 2017 Mar 2;8(15):25334–44. doi: 10.18632/oncotarget.15835 (PMC5421934; doi:10.18632/oncotarget.15835)
Supplement: Supplementary file 2 [file oncotarget-08-25334-s002.doc]

Table S3: The associations between 8 SNPs and the grade 3–4 acute radiation-induced dermatitis

| **SNP** | **Alleles**^a^ | **MAF** | | **N**^b^ | | **Multiplicative** | | **Additive** | | **Dominant** | | **Recessive** | |
| --- | --- | --- | --- | --- | --- | --- | --- | --- | --- | --- | --- | --- | --- |
|  |  | **G3+** | **G0-2** | **G3+** | **G0-2** | **OR(95% CI)** | ***P*** | **OR(95% CI)** | ***P*** | **OR(95% CI)** | ***P*** | **OR(95% CI)** | ***P*** |
| rs9344 | G/A | 0.42 | 0.43 | 1/3/2 | 22/83/43 | 0.95(0.29-3.06) | 0.932 | 1.11(0.28-4.42) | 0.887 | 0.87(0.14-5.47) | 0.883 | 1.84(0.17-20.39) | 0.620 |
| rs3088440 | A/G | 0.08 | 0.11 | 0/1/5 | 2/30/116 | 0.70(0.09-5.60) | 0.736 | 0.59(0.06-5.59) | 0.642 | 0.59(0.06-6.21) | 0.663 | NA | NA |
| rs1059234 | C/T | 0.50 | 0.49 | 2/2/2 | 33/78/37 | 1.06(0.33-3.35) | 0.927 | 0.91(0.26-3.14) | 0.879 | 0.56(0.09-3.58) | 0.536 | 1.45(0.21-9.96) | 0.707 |
| rs12676482 | A/G | 0.08 | 0.12 | 0/1/5 | 1/33/114 | 0.68(0.08-5.41) | 0.712 | 0.55(0.06-5.47) | 0.612 | 0.55(0.06-5.51) | 0.613 | NA | NA |
| rs4755453 | C/G | 0.17 | 0.13 | 0/2/4 | 2/33/113 | 1.40(0.30-6.64) | 0.671 | 2.07(0.36-11.98) | 0.416 | 2.41(0.36-16.25) | 0.365 | NA | NA |
| rs10036748 | C/T | 0.08 | 0.26 | 0/1/5 | 8/60/80 | 0.26(0.03-2.07) | 0.174 | 0.23(0.03-1.92) | 0.174 | 0.21(0.02-1.99) | 0.175 | NA | NA |
| rs2071592 | T/A | 0.42 | 0.46 | 1/3/2 | 36/65/47 | 0.83(0.26-2.67) | 0.753 | 0.76(0.22-2.59) | 0.663 | 0.86(0.13-5.65) | 0.871 | 0.5(0.04-5.52) | 0.568 |
| rs5030437 | A/G | 0.17 | 0.11 | 0/2/4 | 1/31/116 | 1.59(0.33-7.59) | 0.555 | 2.22(0.37-13.25) | 0.381 | 2.55(0.37-17.44) | 0.341 | NA | NA |

^a^ In the order of mutant/wild; ^b^ In the order of mutant homozygote /heterozygote/ wild homozygote;

Abbreviations: RT, radiotherapy; CR, complete remission; MAF, minor allele frequency; G, grade; OR, odds ratio; CI, confidence interval; NA, not applicative.

**Table S4: The associations between 8** SNPs and the grade 3–4 acute radiation-induced oral mucositis

| **SNP** | **Alleles**^a^ | **MAF** | | **N**^b^ | | **Multiplicative** | | **Additive** | | **Dominant** | | **Recessive** | |
| --- | --- | --- | --- | --- | --- | --- | --- | --- | --- | --- | --- | --- | --- |
|  |  | **G3+** | **G0-2** | **G3+** | **G0-2** | **OR(95% CI)** | ***P*** | **OR(95% CI)** | ***P*** | **OR(95% CI)** | ***P*** | **OR(95% CI)** | ***P*** |
| rs9344 | G/A | 0.42 | 0.44 | 7/45/19 | 16/41/26 | 0.91(0.58-1.43) | 0.668 | 0.90(0.54-1.50) | 0.680 | 1.29(0.61-2.71) | 0.502 | 0.46(0.17-1.23) | 0.121 |
| rs3088440 | A/G | 0.09 | 0.13 | 1/11/59 | 1/20/62 | 0.66(0.32-1.36) | 0.259 | 0.64(0.30-1.36) | 0.245 | 0.58(0.26-1.31) | 0.191 | 1.14(0.07-19.30) | 0.927 |
| rs1059234 | C/T | 0.51 | 0.47 | 20/32/19 | 15/48/20 | 1.16(0.74-1.82) | 0.515 | 1.21(0.75-1.95) | 0.424 | 0.90(0.42-1.90) | 0.777 | 1.91(0.87-4.22) | 0.108 |
| rs12676482 | A/G | 0.13 | 0.11 | 1/16/54 | 0/18/65 | 1.19(0.60-2.39) | 0.618 | 1.25(0.59-2.62) | 0.563 | 1.14(0.53-2.49) | 0.735 | NA | NA |
| rs2230926 | C/G | 0.04 | 0.03 | 2/16/53 | 0/19/64 | 1.18(0.33-4.14) | 0.802 | 1.21(0.60-2.44) | 0.590 | 1.09(0.51-2.33) | 0.825 | NA | NA |
| rs10036748 | C/T | 0.23 | 0.27 | 3/26/42 | 5/35/43 | 0.78(0.46-1.32) | 0.356 | 0.79(0.45-1.37) | 0.401 | 0.75(0.39-1.44) | 0.384 | 0.79(0.16-3.77) | 0.765 |
| rs2071592 | T/A | 0.43 | 0.49 | 16/29/26 | 21/39/23 | 0.79(0.50-1.24) | 0.306 | 0.70(0.44-1.10) | 0.124 | 0.53(0.26-1.10) | 0.090 | 0.72(0.33-1.59) | 0.418 |
| rs5030437 | A/G | 0.12 | 0.11 | 1/15/55 | 0/18/65 | 1.12(0.55-2.26) | 0.756 | 1.13(0.54-2.39) | 0.742 | 1.07(0.49-2.34) | 0.864 | NA | NA |

^a^ In the order of mutant/wild; ^b^In the order of mutant homozygote /heterozygote/ wild homozygote;

Abbreviations: RT, radiotherapy; CR, complete remission; MAF, minor allele frequency; G, grade; OR, odds ratio; CI, confidence interval; NA, not applicative.
